# Identification of a novel imprinting mechanism at the X-linked imprinted locus, *X-linked Lymphocyte Regulated 3/4* (*Xlr3/4*)

**DOI:** 10.1186/1756-8935-6-S1-P68

**Published:** 2013-03-18

**Authors:** Sohaib M Qureshi, Michael B Murphy, Seth Kasowitz, Robert J Foley, Michael J O’Neill

**Affiliations:** 1 Genetics and Genomics, University of Connecticut, Storrs, CT 06066, USA

## Background

Genomic imprinting is the epigenetic process by which a subset of genes is expressed from only one allele based on the parent of origin. To date there are approximately 150 confirmed autosomal imprinted loci in the mammalian genome. The epigenetic hallmark of an autosomal imprinted locus is the presence of differential methylation at CpG islands otherwise known as a DMR[1]. Imprint maintenance at autosomal loci is accomplished through specific histone modifications[2]. In 2005, Raefski et.al. found the first X-linked imprinted locus, *Xlr3/4* of which three paralogs, *Xlr3b/4b/4c*, were maternally expressed and paternally silenced in mouse neonatal brain[3]. Here we use a series of techniques to uncover the imprinting mechanism at the *Xlr* locus using known autosomal imprinting mechanisms as a model.

## Materials and methods

Mice were generated that either carried the maternal (39, X^m^) or paternal (39, X^p^) X chromosome. To measure the difference in both primary transcript and mRNA levels of *Xlr3b/4b/4c* between these samples quantitative real time PCR was performed. To search for differential methylation, MeDIP followed by hybridization to an X chromosome tiling array was performed for a global view of methylation at the *Xlr3/4* locus. For confirmation, targeted bisulfite sequencing was performed at CpG islands. Chromatin Immunoprecipitation (ChIP) followed by real time PCR was used to assess differential enrichment of H3K4me3, H3K27me3, H3K9me2, and H3K36me3 at *Xlr3b*. Furthermore, ChIP-Seq was performed to trace RNA Polymerase II (PolII) and H3K36me3 across the Xlr3/4 locus.

## Results

We show that there is no differential methylation at *the Xlr3/4* locus between 39, X^m^ and 39X^P^ samples. Potential candidate regions for differential methylation according to MeDIP assays were disproven by bisulfite sequencing of CpG islands within those regions. Furthermore, all regions assayed at *Xlr3b* were hypermethylated on both alleles. We show that H3K4me3 and H3K27me3 are not enriched at either allele of *Xlr3b* while H3K9me2 is equally enriched on both. We show that the primary transcript of *Xlr3b only* shows imprinted expression at the 3’ end of the gene and this is confirmed by PolII enrichment indicating a stall on the paternal allele during transcriptional elongation. Lastly, H3K36me3 enrichment mirrors that of RNA PolII.

## Conclusions

We show that there is no DMR to govern gene expression at the *Xlr3/4* locus and thus a novel mechanism must exist to regulate the imprint. The current model for this mechanism involves PolII stalling during transcriptional elongation along the paternal allele of *Xlr3b.* On the maternal allele PolII transcribes through the 3’end of the gene unimpeded. While the cause of the stall remains unknown it may involve H3K36me3 histone methyltransferases.
